# Lattice response to the radiation damage of molecular crystals: radiation-induced versus thermal expansivity

**DOI:** 10.1107/S2052520623010636

**Published:** 2024-01-04

**Authors:** Charles J. McMonagle, Chloe A. Fuller, Emanuel Hupf, Lorraine A. Malaspina, Simon Grabowsky, Dmitry Chernyshov

**Affiliations:** a SNBL at ESRF, Grenoble France; b University of Bremen, Department 2 – Biology/Chemistry, Leobener Str. 7, 29359 Bremen, Germany; c University of Bern, Department of Chemistry, Biochemistry and Pharmaceutical Sciences, Freiestrasse 3, 3012 Bern, Switzerland; University of Geneva, Switzerland

**Keywords:** radiation damage, thermal expansion, molecular crystals, disorder

## Abstract

The interaction of intense synchrotron radiation with molecular crystals frequently modifies the crystal structure by breaking bonds, producing fragments and, hence, inducing disorder. Here, a second-rank tensor of radiation-induced lattice strain is proposed to characterize the structural susceptibility to radiation. Quantitative estimates are derived using a linear response approximation from experimental data collected on three materials Hg(NO_3_)_2_(PPh_3_)_2_, Hg(CN)_2_(PPh_3_)_2_ and BiPh_3_ [PPh_3_ = triphenylphosphine, P(C_6_H_5_)_3_; Ph = phenyl, C_6_H_5_], and are compared with the corresponding thermal expansivities. The associated eigenvalues and eigenvectors show that the two tensors are not the same and therefore probe truly different structural responses. The tensor of radiative expansion serves as a measure of the susceptibility of crystal structures to radiation damage.

## Abstract 2

The interaction of intense synchrotron radiation with molecular crystals frequently modifies the crystal structure by inducing disorder. A second-rank tensor of radiation-induced lattice strain is proposed to characterize lattice susceptibility to the radiation.

## 1. Introduction

The damaging effects of X-ray radiation have long been known and have been extensively investigated in protein crystallography, with the main aim to develop experimental strategies that minimize the damage (Garman, 2010 ▸; Bourenkov & Popov, 2010 ▸). The same level of attention and understanding is not yet present in small molecule crystallography, but radiation damage is becoming more and more frequently observed with diffraction experiments at modern synchrotron radiation sources. The damage manifests as structural disorder in the crystal that suppresses scattering at high angles and can heat the crystal and/or modify the equilibrium between various polymorphic modifications. Prominent recent examples are Lawrence Bright *et al.* (2021 ▸), Bogdanov *et al.* (2021 ▸), Christensen *et al.* (2019 ▸), Coates *et al.* (2021 ▸), Chernyshov *et al.* (2022 ▸), and Grzechnik *et al.* (2023 ▸). Moreover, X-ray radiation may strongly affect physical properties such as electronic configurations, colour, compressibility, thermal expansion, stability of catalysts, and many others (Ishibashi *et al.*, 2002 ▸; Coates *et al.*, 2021 ▸; Collings & Hanfland, 2022 ▸; Fernando *et al.*, 2021 ▸; Wu *et al.*, 2014 ▸). Understanding and controlling these radiation effects might, therefore, offer new possibilities to deliberately tune physical properties of crystalline small molecule materials, and to use ‘X-rays as a design tool in their own right’ (Coates *et al.*, 2021 ▸).

Quantification of radiation effects can be challenging. It involves measurements of the incoming flux, absorbing properties of the materials, shape and intensity distribution parameters of the beam, and the shape and size of the crystal; the option to reliably determine those parameters is not always present at synchrotron beamlines. It can be particularly difficult for many *in-situ* and *operando* experiments, if possible at all.

On the other hand, the structural response to radiation can be seen as a material property that quantifies the structural susceptibility to radiation. Such a property is defined by the chemical composition, the inter- and intra-molecular bonding, the intensity and wavelength of the penetrating radiation, and the accumulated dose. The kinetics may also play an important role, in particular for finite rates of the structural changes induced by radiation. At the onset of the radiation damage, when the concentration of radiation-induced defects is still low, the change in volume of the averaged unit cell might serve for quantification purposes. For proteins a linear correlation between volume and dose was noted and this was found to be strongly material dependent (Ravelli *et al.*, 2002 ▸).

To facilitate comparisons between different systems, a volume X-ray expansion coefficient was introduced in Coates *et al.* (2021 ▸). Here we further develop this idea, providing a phenomenological parameterization of the induced lattice changes with a second-rank tensor to characterize the radiation expansivity. We report on the observed radiation effects in three low-symmetry molecular crystals, [Hg(NO_3_)_2_(PPh_3_)_2_, Hg(CN)_2_(PPh_3_)_2_ and BiPh_3_ (PPh_3_ = triphenylphosphine, P(C_6_H_5_)_3_; Ph = phenyl, C_6_H_5_)] and contrast this to the thermal expansivity derived from low-dose temperature-dependent measurements.

## 2. Experiment

The compound BiPh_3_ was obtained commercially and recrystallized from acetonitrile according to Hawley & Ferguson (1968 ▸). Both Hg(NO_3_)_2_(PPh_3_)_2_ and Hg(CN)_2_(PPh_3_)_2_ were synthesized by mixing and stirring the commercially obtained PPh_3_ and mercury salts Hg(NO_3_)_2_/Hg(CN)_2_ in stoichiometric ratios in a dichloromethane solution. After evaporation of the solvent the crude mercury compounds were recrystallized from ethanol according to Buergi *et al.* (1982 ▸). Crystals of ∼100 microns in size and of nearly isotropic shape were selected for the measurements. Two to four crystals of each compound were tested as a function of time during dose accumulation; the high dose behaviour depends on the X-ray energy and intensity and is challenging to reproduce in a controlled fashion. We therefore focus on a low-dose structural response.

Single-crystal Bragg scattering data were collected at the BM01 end station of the Swiss–Norwegian Beamlines on the Pilatus@SNBL diffractometer (Dyadkin *et al.*, 2016 ▸) using a four-bunch 30-mA mode of the synchrotron beam (the default mode is 200 mA multibunch). The wavelength was set to λ = 0.7277 Å, giving a flux of 7.4 × 10^7^ photons per second at the sample position. This is significantly below the peak flux available on BM01 and up to five orders of magnitude lower than the flux at ESRF protein beamlines. This low flux beam was specifically used to reduce the dose accumulation rate and slow the structural relaxations associated with radiation effects and mitigate possible kinetics effects.

Each dataset was measured with an omega scan, angular step = 0.5°, in the 0–360° angular range. These data were used for structural analysis and were processed with the *CrysAlisPro* software (Rigaku, 2015 ▸). The structures were solved with *SHELXT* (Sheldrick, 2015*b* ▸), and then refined with *SHELXL* (Sheldrick, 2015*a* ▸), and we employed the Seq-Shel tool for sequential processing and structure refinement (Chernyshov *et al.*, 2019 ▸).

The total dose of radiation experienced by the sample per data acquisition can be estimated using *RADDOSE-3D*, a program first developed for single-crystal macromolecular samples, and more recently adapted to quantify dose rates for small molecule crystallography and for small-angle scattering (SAXS) measurements (Zeldin *et al.*, 2013 ▸; Bury *et al.*, 2018 ▸; Brooks-Bartlett *et al.*, 2017 ▸). *RADDOSE-3D* considers the crystal (shape, size, unit cell, composition), the beam (size, profile, energy, and flux), and how the data were collected (angular wedge and total exposure time). For dose accumulation collections, the strategies were consistent with a Gaussian beam profile, 310.00 by 300.00 FWHM (*x* by *y*), and a flux of 7.4 × 10^7^ photons per second at 17.04 keV (λ = 0.7277 Å), and data collection for a total of 90.0 s from 0.0 to 360.0°. This gives an estimated average dose on the whole crystal per data collection for Hg(NO_3_)_2_(PPh_3_)_2_, Hg(CN)_2_(PPh_3_)_2_ and BiPh_3_ of 466, 503 and 750 Gy, respectively.

To extract thermal expansion effects, data were collected during a 16-bunch 70-mA mode with a wavelength of λ = 0.6052 Å. A shorter wavelength was selected to reduce the interaction energy of the X-rays with the samples and a strong filter (100 µm of Cu, 5% transmission) was used to avoid or minimize possible radiation effects giving a flux at the sample of 2.16 × 10^6^ photons per second. The data collection strategy was optimized to reduce exposure by limiting the data collection to a small angular range that is sufficient for a reliable determination of the unit-cell dimensions with collections made in the 100–300 K range using a Cryostream 700+ open flow nitrogen blower.

## 3. Results and discussion

### 3.1. Measured expansion behaviour

**Figure 1:**
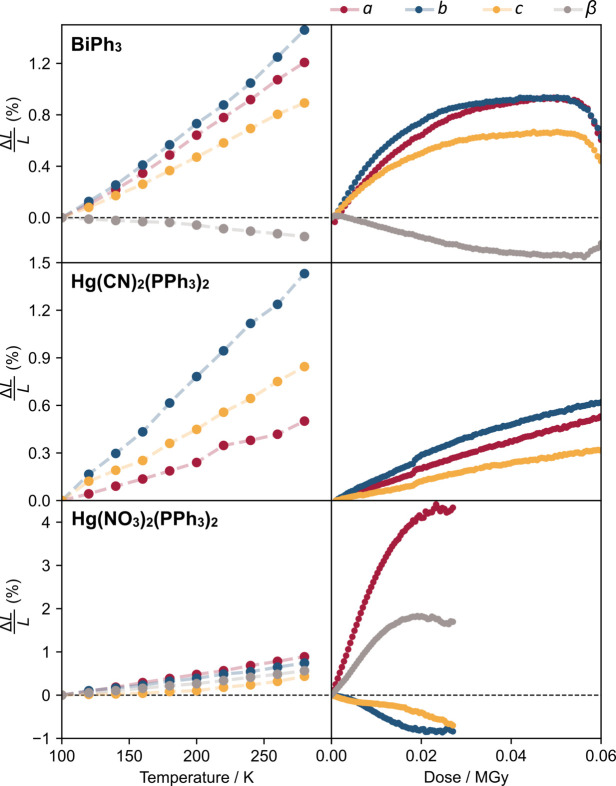
Expansion of unit-cell parameters as a function of temperature and dose. Unit-cell parameters are given as percentage differences relative to their values at 100 K and zero dose for easier comparison.

A comparison of unit-cell dimensions as a function of temperature (LHS) and of accumulated dose (RHS) is shown in Fig. 1 ▸. In the low-dose region, BiPh_3_ shows comparable expansion behaviour on heating and with dose, with the directions and relative magnitudes of all axes being consistent. As dose increases, the lattice expansion plateaus, and a further increase results in a lattice contraction before the crystallinity is completely lost. In contrast, for Hg(CN)_2_(PPh_3_)_2_, the radiation expansivity is approximately linear throughout the tested dose range, and is considerably lower than that due to thermal effects. The *b*-axis shows the largest strain in both cases while *a* and *c* differ for radiation and temperature. For Hg(NO_3_)_2_(PPh_3_)_2_, the difference is marked, with the relative variation of the *a*-axis under irradiation being almost four times larger than that induced by increasing the temperature from 100 to 280 K. The *b*- and *c*-axes have the opposite sign for radiation and temperature response leading to a negative area dose expansivity analogous to negative area compressibility (Cairns & Goodwin, 2015 ▸).

The simplest way to parameterize the expansion shown in Fig. 1 ▸ would be to quote the volume expansion. However, for non-cubic systems, this belies the anisotropic nature of the expansion. A more complete description can be gained using a second-rank strain tensor (Belousov & Filatov, 2007 ▸), as is commonly done to quantify the lattice response to temperature or pressure. We can introduce an analogous second-rank tensor of radiative expansion coefficients, α_*ij*_, which relates radiation-induced deformations (strains, ɛ_*ij*_) to the accumulated dose, *D*: 

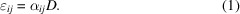

This implies the following dose dependence for any lattice vector **d**


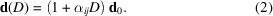

**Figure 2:**
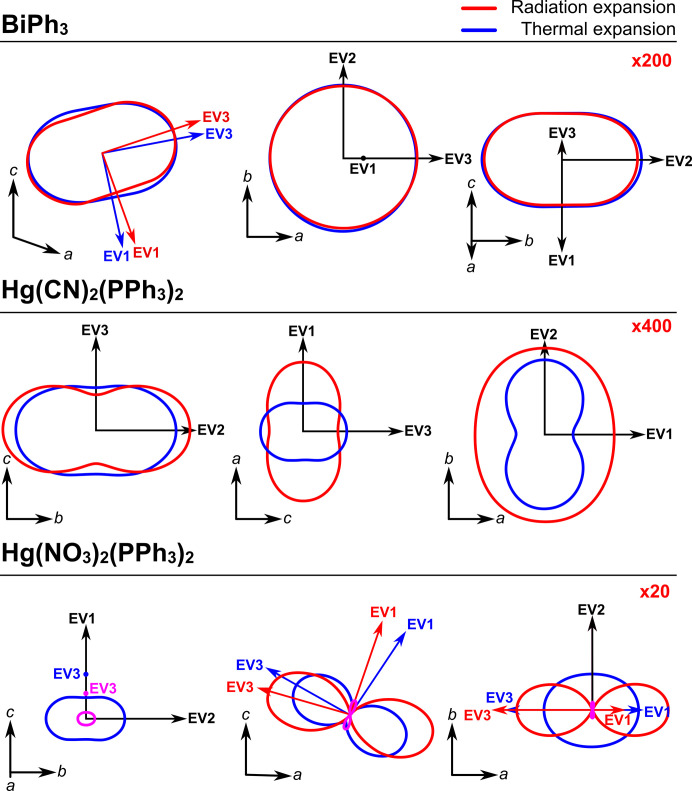
Expansion indicatrices of the three compounds. Each panel shows three sections of the indicatrix, viewed along each of the Cartesian coordinates, with the corresponding unit cell vectors and expansion eigenvectors (EV) labelled. Blue sections show thermal expansion and red the expansion due to radiation. The pink lines indicate negative expansion induced by radiation. Where the thermal and radiation expansion share the same eigenvectors, these are shown in black, otherwise they are shown in blue and red, respectively. To make the thermal expansion (in K^−1^) and radiation expansion (in Gy^−1^) a comparable size in the diagram, the radiation expansion has been multiplied by the figure given in red in the top right of each panel.

Since this is directly comparable to thermal expansion, the transformation of the crystallographic coordinate systems to an orthogonalized physical coordinate system, and the calculation of tensor eigenvalues and eigenvectors can all be performed with existing software. Such algorithms are reported in detail by Belousov & Filatov (2007 ▸), Cliffe & Goodwin (2012 ▸), Bubnova *et al.* (2013 ▸), Jones *et al.* (2013 ▸) and Langreiter & Kahlenberg (2015 ▸). Such software allows the calculated expansivity tensor to be represented by plotting the expansion coefficients as a function of direction resulting in a 3D surface of the expansivity indicatrix (Belousov & Filatov, 2007 ▸; Cliffe & Goodwin, 2012 ▸). 2D sections of 3D indicatrix surfaces for the thermal and radiation-induced cell expansion are shown in Fig. 2 ▸, and the full tensors are given in Tables S1 and S2. These were calculated with the program *TEV* (Langreiter & Kahlenberg, 2015 ▸) using only the initial collections for each crystal where the response was linear. At a glance, indicatrix plots expand our understanding of the differences in response to temperature and dose. They give the magnitudes, sign, and directionality of the expansions and how these differ. This is particularly important for low-symmetry systems like the monoclinic Hg(NO_3_)_2_(PPh_3_)_2_ and BiPh_3_, where the principle directions of thermal and radiative expansion are not equivalent to each other, or to the unit cell axes, or even to structural motifs. Figs. S1, S2 and S3 show overlays of the crystal structure projections with sections of the expansivity indicatrices, showing the challenge in predicting/rationalizing these effects.

These three compounds were selected according the following criteria. Firstly they are all high quality single crystals that are also molecular compounds where the heavy element, understood to be responsible for the radiation sensitivity, is isolated from other heavy elements, *i.e.* diluted in a surrounding of light elements. BiPh_3_ is a metal–organic compound (Bi—C bonds) whereas the two Hg-containing compounds are not (no Hg—C bonds). They are also compounds that through testing have been shown to display beam damage behaviour as a response to X-ray irradiation. For a better understanding of the origin of these defects local probe techniques would be more appropriate rather than the average structure methods employed here.

**Figure 3:**
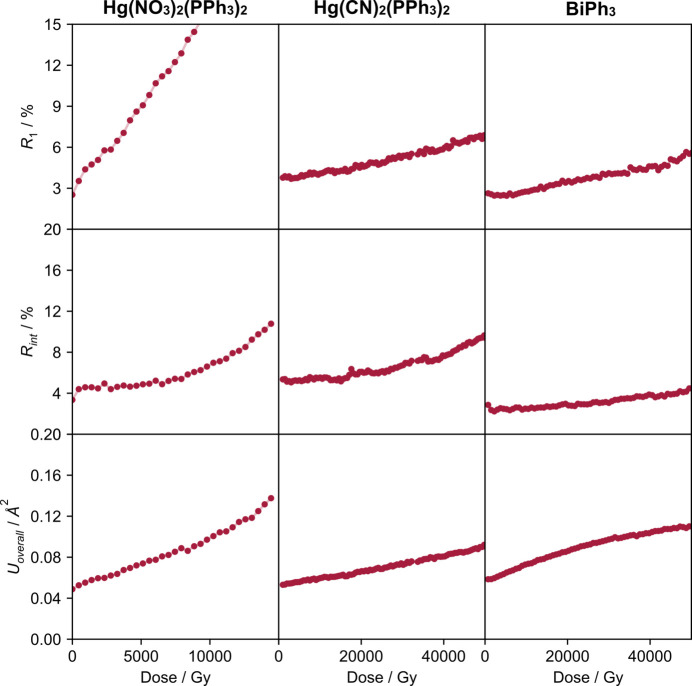
Evolution of the crystallographic parameters *R*_1_, *R*_int_ and *U*_overall_ following radiation damage with dose for the three compounds studied. Note the change in dose scale for Hg(NO_3_)_2_(PPh_3_)_2_.

As expected, dose accumulation is accompanied by a degradation of crystal quality that is reflected in the *R*-factors and overall atomic displacement parameters in the structural refinements (Fig. 3 ▸). In general, these descriptors correlate with radiative expansion: Hg(NO_3_)_2_(PPh_3_)_2_ has the highest volume coefficient and the fastest growth of *R*_1_, *R*_int_ and *U*_overall_. However, the two other materials show similar increases in all three metrics despite having quite different radiation expansion behaviour. The *R*-factors for Hg(CN)_2_(PPh_3_)_2_ mirror its fairly linear increase in lattice parameters across the measured dose range, whereas for BiPh_3_, the unit-cell parameters increase more sharply before plateauing, something that is not evident in the *R*-factors.

### 3.2. Mathematical comparison between thermal and radiation expansion

Since radiation and temperature both produce expansions of the unit cell it might be natural to assume that the underlying mechanisms are linked, similar to the supposed inverse relationship between lattice response to temperature and pressure [*i.e.* rule by Hazen and Finger (1984 ▸)]. Both thermal and radiative expansion arise from changes in bonding interactions and are therefore functions of the elastic properties of the material. However, the significant differences in the lattice responses to the two stimuli, illustrated by Figs. 1 ▸ and 2 ▸, indicate that the two tensors probe truly different structural changes, which are unlikely to be correlated. This is consistent with Coates *et al.* (2021 ▸) and mirrors the findings of Kaźmierczak *et al.* (2021 ▸) which show that temperature and pressure invoke very different responses in a significant portion of materials.

Within the Grüneisen model, the anisotropic tensor of thermal expansion is given by (Kamencek *et al.*, 2022 ▸):

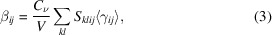

where *S*_*klij*_ is the elastic compliance tensor, *C*_ν_ is the phonon heat capacity at constant configuration and *V* is the volume. 〈γ_*ij*_〉 is the mean Grüneisen tensor (Ritz *et al.*, 2019 ▸) that serves as a measure of anharmonicity of phonon modes with respect to anisotropic strain components:

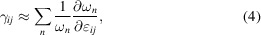

where ω_*n*_ is the frequency of phonon mode *n*. The average 〈γ_*ij*_〉 is calculated with respect to the contribution of individual modes into *C*_ν_. For example, in the case of orthorhombic symmetry [as in Hg(CN)_2_(PPh_3_)_2_] the thermal expansion along the *a*-direction is

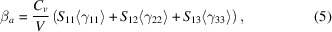

where the elements of the compliance tensor are given in the Voigt notation. Therefore the anisotropy of thermal expansion is not a sole function of elastic properties, it also depends on the anisotropy of anharmonic potential expressed via the Grüneisen tensor.

To qualify the relationship between lattice strain and radiation-induced defects, we can consider the following potential, *Y*, 

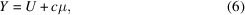

where the energy of the host lattice, *U*, is separated from the chemical potential associated with a defect formation, μ, multiplied by the concentration of these defects, *c*. It is assumed that the concentration of defects is sufficiently low to stay in the linear response regime. Likewise, changes in entropy, including vibrational entropy and internal energy, induced by the defects are also ignored.

Neglecting thermal effects, the equilibrium condition with respect to the lattice deformations ɛ_*ij*_ can be written as 

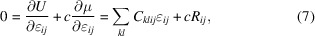

where *C*_*klij*_ represents the elements of the fourth-rank tensor of elastic constants, and *R*_*ij*_ is a second-rank tensor of derivatives of chemical potential for a radiation-induced defect with respect to the deformations of the host structure. Correspondingly, the lattice deformations (or radiation-induced expansivity) is given by

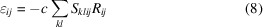

where *S*_*klij*_ is a fourth-rank tensor of elastic compliance. A second-rank tensor *R*_*ij*_ serves as a measure of lattice susceptibility to the defect formation in the host lattice. The elastic response of the crystal lattice comprises both the elastic anisotropy of the host lattice, and the anisotropy of defect-related chemical potential with respect to the deformations of the host lattice. The similarity between equations (3) and (8) is obvious.

A more rigorous way to rationalize the anisotropy of the deformations assumes that the radiation expansivity is mostly defined by the elastic properties of the non-damaged part of the crystal. Therefore, the effect of radiation-induced defects may be seen as an internal pressure. A suitable solid state elastic theory was developed for spin crossover solids where a change of spin state in a molecular complex was considered as a defect (Willenbacher & Spiering, 1988 ▸); here we briefly recall the basic ideas of the approach relevant to our problem.

An anisotropic defect can be seen as an elastic dipole characterized by a six-component elastic dipole tensor **P**. Insertion of a defect into an anisotropic elastic media results in a deformation field, represented by the second-rank tensor ɛ_*ij*_, and proportional to **P**. If there are few equivalent positions/orientations of the defect in the average structure, ɛ_*ij*_ represents the averaged deformation field. The anisotropy of radiative expansivity depends on the elastic properties of the defect-free media [via Lamé coefficients or bulk modulus and Poisson ratio given in Willenbacher & Spiering (1988 ▸)] and the elastic dipole tensor associated with radiation-induced defects. Despite the oversimplified and qualitative considerations given here, there is no physical reason for anisotropy of thermal and radiation expansivity to be the same. Assuming that concentration of defects, *c*, is proportional to the absorbed dose, *c* = *kD*, and neglecting interactions between defects, for any lattice vector **d** the dose dependence is expected to be:

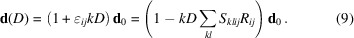




## 4. Conclusions

Radiative and thermal expansion can both be expressed as a second-rank tensor quantified by expansion coefficients and visualized by expansivity indicatrices. The radiative expansion may therefore be seen as a measure of the material’s susceptibility to radiation impact. Quality and self-consistency of diffraction data normally degrades under intense irradiation such as at synchrotrons and *R*-factors increase. The other frequently seen effect is a suppression of high-angle diffraction intensities which manifests as an increase of the overall displacement parameter. One needs quite a complete and redundant data set to get reliable estimates of *R*-factors and *U*_overall_, and this gets more difficult if radiation damage becomes significant. In contrast, unit-cell dimensions can be estimated from a relatively small subset of single crystal reflections, or a single powder pattern, and can serve as an indicator of accumulated dose providing that a second-rank tensor of radiative expansion coefficients is known.

At the level of elastic response, radiative expansivity relates to deformations induced by insertion of anisotropic defects while thermal expansion is defined by anisotropy of elastic stiffness and Grüneisen parameters. Structural deformations induced by temperature (‘phonon pressure’) and radiation damage (‘defect pressure’) are not necessarily correlated with each other. For two out of three materials studied in the present work, phonon and defect pressure induced deformations are directionally different from each other. To determine the exact nature of the defects one would want to look at the local and not an average structure. It seems that different bonds and inter-molecular contacts are affected by anharmonic phonons and radiation-induced defects. Therefore, thermal and radiative expansion have different underlying structural mechanisms.

The collection strategy presented here was specifically chosen to induce the effects of radiation in a slow and controlled fashion to quantify the radiative expansion coefficient. In a more typical synchrotron experiment it would be extremely easy to accumulate and hugely exceed the total accumulated doses described here, 0.06 MGy, in a single experiment or image. For example, if the same collection strategy as described here was collected using the unfiltered beam of BM01, a single collection would account for an average dose of 1.2 MGy. In this case it would not be possible to extract the effects of radiation. Strategies to reduce dose effects would be to use lower fluxes, lower temperatures, and shorter collection times. Extra caution must be made when making multiple measurements on the same sample as is the case for variable temperature studies. Deconvoluting the thermal expansivity in the presence of radiative expansion is not trivial. Good experimental design and judicious use of high flux sources is, as always, recommended. 

## Supplementary Material

Crystal structure: contains datablock(s) global, Bi_Ph3, Hg_CN2, Hg_NO3. DOI: 10.1107/S2052520623010636/ra5138sup1.cif

Structure factors: contains datablock(s) Bi_Ph3. DOI: 10.1107/S2052520623010636/ra5138Bi_Ph3sup2.hkl

Structure factors: contains datablock(s) Hg_CN2. DOI: 10.1107/S2052520623010636/ra5138Hg_CN2sup3.hkl

Structure factors: contains datablock(s) Hg_NO3. DOI: 10.1107/S2052520623010636/ra5138Hg_NO3sup4.hkl

Supplementary figures and tables. DOI: 10.1107/S2052520623010636/ra5138sup5.pdf

CCDC references: 2320616, 2320617, 2320618
