# Supplementary material for: Lattice response to the radiation damage of molecular crystals: radiation-induced versus thermal expansivity
Source: Acta Crystallogr B Struct Sci Cryst Eng Mater. 2024 Jan 4;80(Pt 1):13–8. doi: 10.1107/S2052520623010636 (PMC10848411; doi:10.1107/S2052520623010636)
Supplement: Supplementary file 5 [file b-80-00013-sup5.pdf]

STRUCTURAL SCIENCE
CRYSTAL ENGINEERING
MATERIALS

Volume 80 (2024)

Supporting information for article:

Lattice response to the radiation damage of molecular crystals: radiation-induced versus thermal expansivity

Charles J. McMonagle, Chloe A. Fuller, Emanuel Hupf, Lorraine A. Malaspina, Simon Grabowsky and Dmitry Chernyshov

BiPh₃

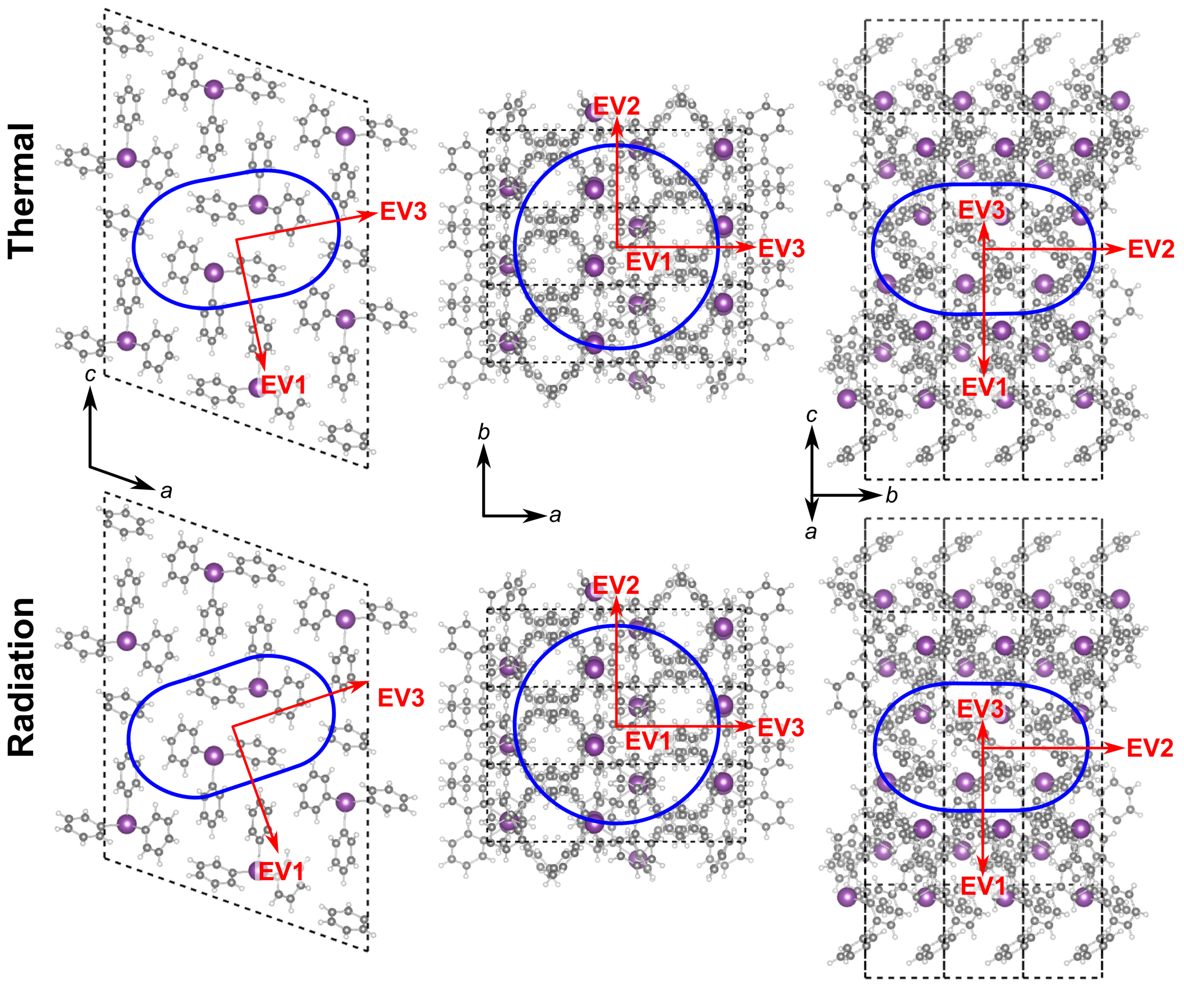

Figure S1: Overlays of the crystal structure of $BiPh_3$ viewed along various axes and the corresponding section of the thermal and radiative expansivity indicatrices. Blue lines indicate a positive expansion and black, a negative expansion. The eigenvectors of the expansion are shown in red.

$Hg(CN)_2(PPh_3)_2$

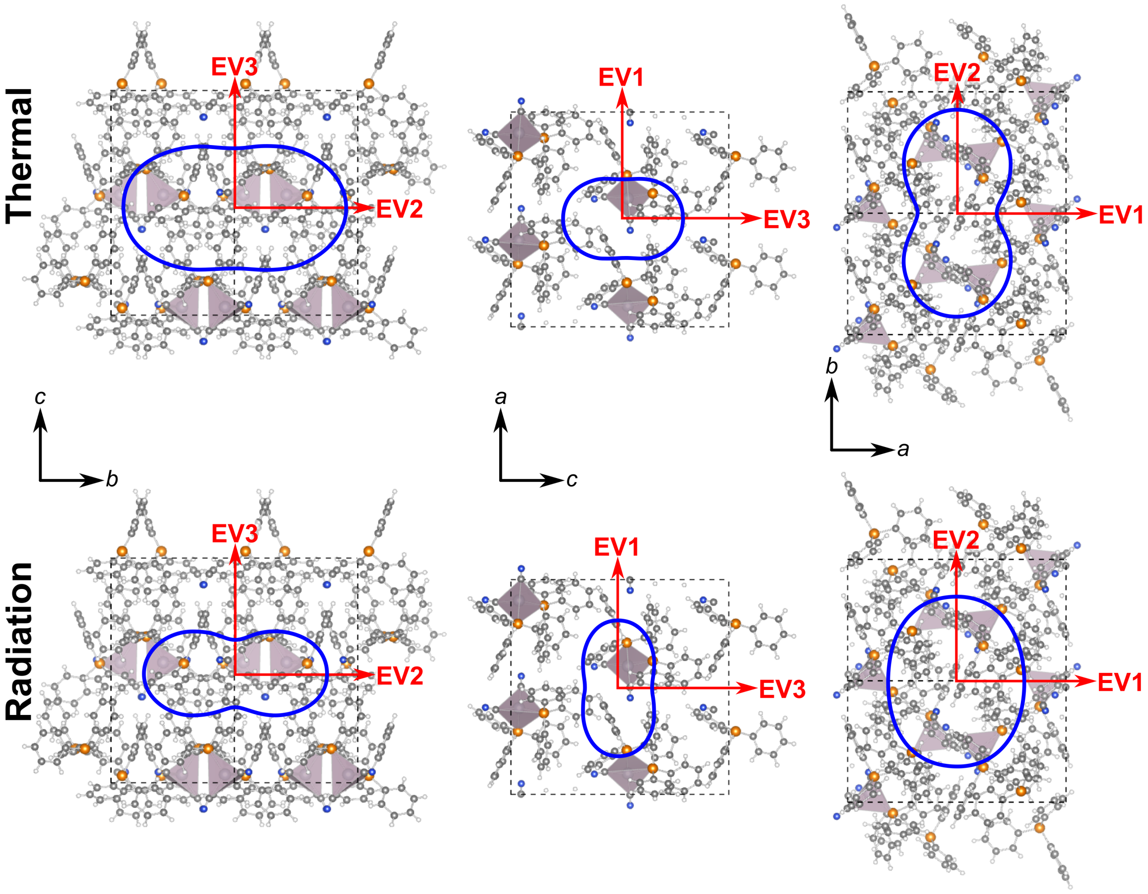

Figure S2: Overlays of the crystal structure of $Hg(CN)_2(PPh_3)_2$ viewed along various axes and the corresponding section of the thermal and radiative expansivity indicatrices. Blue lines indicate a positive expansion and black, a negative expansion. The eigenvectors of the expansion are shown in red.

$Hg(NO_3)_2(PPh_3)_2$

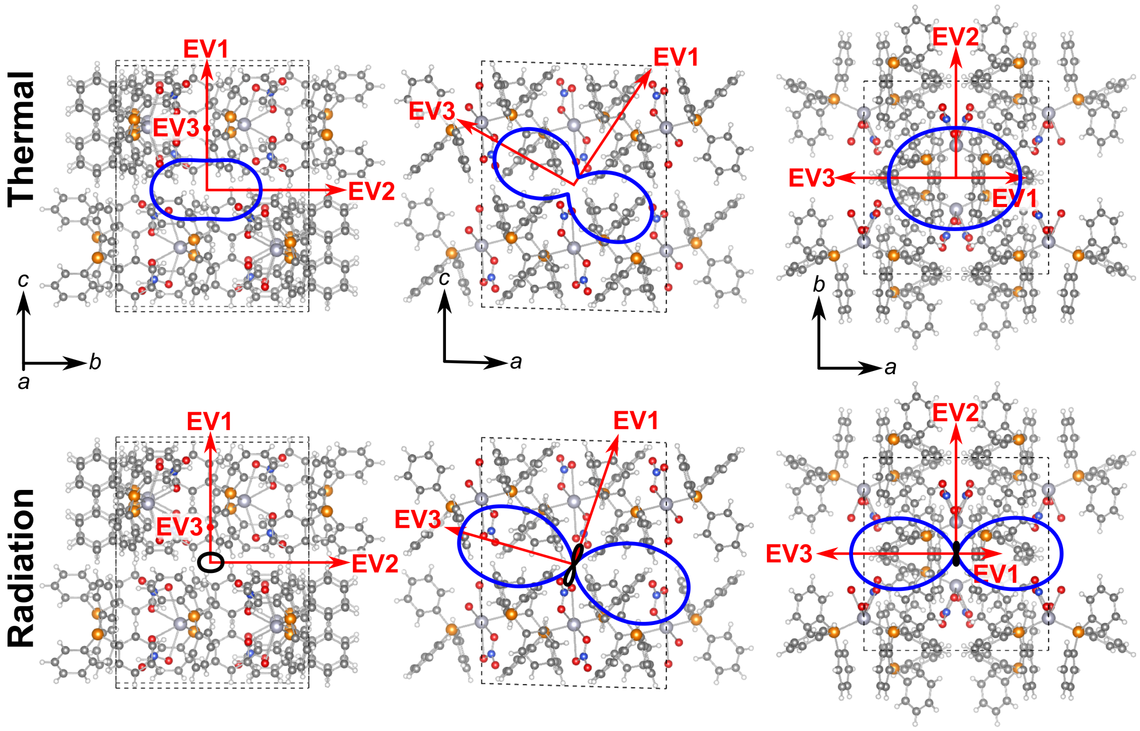

Figure S3: Overlays of the crystal structure of $Hg(NO_3)_2(PPh_3)_2$ viewed along various axes and the corresponding section of the thermal and radiative expansivity indicatrices. Blue lines indicate a positive expansion and black, a negative expansion. The eigenvectors of the expansion are shown in red.

Thermal and radiative expansion tensors

Table S1: Thermal expansion tensor components for the three materials.

Compound	Thermal expansion coefficients / MK^{-1}					
	α_{11}	α_{22}	α_{33}	α_{12}	α_{13}	α_{23}
$Hg(NO_3)_2(PPh_3)_2$	48	40	23	0	-25	0
$Hg(CN)_2(PPh_3)_2$	28	79	46	0	0	0
$BiPh_3$	74	80	51	0	5	0

Table S2: Radiative expansion tensor components for the three materials.

Compound	Radiative expansion coefficients / MGy^{-1}					
	α_{11}	α_{22}	α_{33}	α_{12}	α_{13}	α_{23}
$Hg(NO_3)_2(PPh_3)_2$	2.8	-0.33	-0.26	0	-1.1	0
$Hg(CN)_2(PPh_3)_2$	0.10	0.13	0.05	0	0	0
$BiPh_3$	0.46	0.48	0.31	0	0.06	0
